# Perspectives on Ovarian Cancer 1809 to 2022 and Beyond

**DOI:** 10.3390/diagnostics12040791

**Published:** 2022-03-24

**Authors:** Frank G. Lawton, Edward J. Pavlik

**Affiliations:** 1 Gynaecological Cancer Surgeon South East London Gynaecological Cancer Centre, Guy’s and St Thomas’ NHS Trust, London SE1 7EH, UK; fglawtongyn@aol.com; 2 Division of Gynecologic Oncology, Department of Obstetrics and Gynecology, University of Kentucky Chandler Medical Center-Markey Cancer Center, Lexington, KY 40536, USA

**Keywords:** ovarian cancer, surgical debulking, surgery at relapse, interval debulking, neoadjuvant chemotherapy, ultra-radical surgery, genomics, liquid biopsy, PARP inhibitors, immunotherapy, CAR T cell therapies, HIPEC, prevention

## Abstract

Unlike many other malignancies, overall survival for women with epithelial ovarian cancer has improved only modestly over the last half-century. The perspectives presented here detail the views of a gynecologic oncologist looking back and the view of the academic editor looking forward. Surgical beginnings in 1809 are merged with genomics, surgical advances, and precision therapy at present and for the future. Presentations in this special issue focus on factors related to the diagnosis of ovarian cancer: (1) markers for the preoperative assessment of primary and metastatic ovarian tumors, (2) demonstrations of the presence of pelvic fluid in ultrasound studies of ovarian malignancies, (3) the effects of age, menopausal status, and body habitus on ovarian visualization, (4) the ability of OVA1 to detect ovarian cancers when Ca125 was not informative, (5) the detection of tumor-specific changes in cell adhesion molecules by tissue-based staining, (6) presentation of a high discrimination model for ovarian cancer using IOTA Simple Rules and CA125, (7) review of low-grade serous carcinoma of the ovary, and (8) a comprehensive case report on ovarian carcinosarcoma.

## 1. Introduction and Views from a Gynecologic Oncologist

The genesis of ovariectomy for ovarian tumors was when Jane Todd Crawford became the first person to undergo such surgery on Christmas Day 1809 in Danville, Kentucky. The surgeon Ephraim McDowell removed a 22 lb mass under an ‘anaesthetic’ of oral opium with a number of attendants holding her still in a 25 min operation! Discharged in January 1810, she lived for another 32 years [1]. The short operating time and her survival, suggest that the tumor was either benign or a borderline tumor or, if malignant, confined to one ovary. Since then, surgery has been preeminent in the treatment of ovarian malignancies: medical or radiation oncologists being unlikely to receive a referral without, at least, a biopsy-proven diagnosis. It became clear, early in the last century, that most ovarian cancers were not curable by surgery alone, and this conclusion remains valid to the present day. Management would consist of laparotomy, a biopsy with perhaps an attempt at more extensive intervention, then radiotherapy. However, despite improvements in surgery and radiotherapy techniques, patient outcome was disappointing; the five-year survival rate, having improved only from 29 percent (1950–1959) to 32 percent (1965–1969). At the same time, interest in postoperative chemotherapy was emerging. A palliative effect had been indicated through a reduction in the incidence of recurrent ascites; moreover, expectations for a potential for ‘cure’ arose from an early study with the alkylating agent melphalan, in which 13 patients ‘had such an unusual good response that laparotomy was performed to evaluate if an inoperable tumor had become removable [and] to evaluate the need for additional therapy. In each of the 13 patients, no tumor was found and chemotherapy was discontinued’. At a later follow-up, only two patients had developed a recurrence [2].

## 2. ‘Debulking’ Surgery

Many studies have shown that patients beginning treatment with a small volume of post-surgical disease will have a longer time before clinical recurrence is documented than patients with a bulkier residuum, thus linking minimal residual disease to better outcomes. These observations had an impact on post-surgical treatment such that the amount of disease before the initiation of chemotherapy would be considered a factor in stratification or even an exclusion criterion in most randomized studies of post-surgical treatment.

In the mid-1970s, the goal for ‘leaving as little as possible’ was interpreted as individual tumor masses of 1.6 cm, which in practice became 2 cm or less. However, leaving macroscopic disease in situ, and considering this to be ‘optimal’ proved to be inadequate. Optimal is now accepted as leaving no macroscopic residuum [R0].

It was believed, without any proof or evidence from randomized trials, that surgery could overcome any adverse biological factors leading to a subsequent spread of tumor burden and that a minimal tumor burden would be more likely to be chemosensitive. In other words, ‘The less tumor the better’!

A population-based study in 2008 reported that cure rates for ovarian cancer had improved from 12% to 14%, far inferior to colorectal (29% to 47%) and testicular cancer (23% to 81%) [3]. In the USA, from 1973–1999, the five-year case-fatality rate (the proportion of patients who died from their cancer) fell by 7.5%, whilst the 12-year rate fell by only 1.2% [4]. The authors linked the five-year decrease with the introduction of platinum and taxanes into clinical practice. The effect of surgery was remarkably absent in both studies. 

Achieving R0 and the likely increased morbidity and mortality associated with more aggressive surgery has been the subject of studies proposed over the last few years; however, no prospective randomized study has ever been completed to determine whether an ‘optimal’ surgical outcome is due to surgical endeavor, skill, and determination, or because some ovarian tumors are biologically less aggressive, are less widespread, or do not have upper abdominal, splenic, diaphragmatic involvement at presentation, and so are easier to extirpate. 

## 3. Surgery at Relapse

Treatment at relapse has focused on further chemotherapy, surgery being indicated in relieving symptoms (bowel by-pass, stoma formation, etc.), but the place of further surgery has been investigated in two randomized studies. The question of surgery remains the same: if it can extirpate all visible recurrence, will this be shown to have a positive effect on further tumor recurrence, progression-free interval, or survival? 

Gynecologic Oncology Group study 213, in which a complete resection of disease was thought feasible, randomized patients to chemotherapy alone or to secondary surgical cytoreduction and chemotherapy [5]. No difference was seen in either progression-free or overall survival. A sub-analysis of those patients with a single site of disease [nodal, splenic, etc.] also did not show improvement due to surgery.

A second trial, DESKTOP III, however, showed an improvement in both progression-free-interval (18.4 vs. 14 months) and overall survival (53.7 vs. 46.2 months) in favor of surgical intervention [6]. The authors did report that such an approach should be reserved for only those patients for whom complete resection was considered to be likely and that secondary debulking should only be considered for patients with a single site of disease and good performance status. Re-operation for patients with platinum-resistant disease, miliary disease, or ascites was not recommended [7]. Attempts have been made to explain these conflicting results [5]. It is possible that the biology of recurrent disease may be different from that at initial presentation, but it is widely felt that the patients who can benefit from surgery at relapse will be those who have had a relatively long disease-free interval after completion of platinum-containing therapy (at least six but preferably 12 months), with isolated small volumes of recurrence and no or minimal ascites. 

The responses to chemotherapy by other cancers (i.e., breast) have suggested that if the bulk of the tumor at presentation can be reduced by neoadjuvant chemotherapy, initial surgery may be less morbid in terms of the extent of resection, and subsequent chemotherapy more effective, thus improving outcomes. 

### Neoadjuvant Chemotherapy (NACT) and Interval Debulking Surgery (IDS)

Two studies, CHORUS [8] and EORTC 55971 [9], compared ‘upfront’ debulking surgery followed by chemotherapy with NACT/IDS. Outcomes in terms of median survival differed between the two studies with median survival in CHORUS at 23.6 months, versus 30.2 months for EORTC 55971 (*p* = 0.004), but overall, no survival difference was noted between patients who underwent neoadjuvant chemotherapy compared with upfront debulking surgery (27.6 months and 26.9 months *p* = 0.586). 

It was noted that women with stage IV ovarian cancer had significantly better outcomes with neoadjuvant chemotherapy compared with upfront debulking surgery with a median progression-free survival of 10.6 months compared with 9.7 months (*p* = 0.049) and a median overall survival of 24.3 months compared with 21.2 months (*p* = 0.048) [9]. 

A large study using the US National Cancer Database compared the outcome of nearly 3000 patients scheduled to receive NACT/IDS with an equal number, matched for age, stage, grade, and histology undergoing primary surgery followed by chemotherapy [10]. Median survival was shorter for the NACT group (32.1 vs. 37.3 months), but the authors did suggest that differences in performance status between the groups might have affected the outcome because just over a quarter of patients in the NACT group did not undergo surgery, while 15% in the primary surgery group did not receive postoperative chemotherapy.

## 4. Ultra-Radical Surgery

The extent of disease in the upper abdomen—diaphragmatic, hepatic, splenic, pericardiac nodal involvement—presents a considerable barrier to achieving R0. There is the possibility of increased morbidity and possible mortality that would accompany extensive, extirpative surgery in the upper abdomen. However, gynecological oncologists have undertaken extensive, ultra-radical procedures, sometimes in the absence of data from randomized trials. 

By incorporating procedures such as diaphragm surgery, splenectomy, distal pancreatectomy, partial liver resection, cholecystectomy, and porta hepatis tumor resection, the proportion of patients achieving optimal cytoreduction can be doubled (11% to 27%) without significantly increasing postoperative complications resulting in a median overall survival (OS) time of 54 months with ultra-radical surgery vs. 43 months without ultra-radical surgery, *p* = 0.03) [11]. Harter et al. reported similarly improved outcomes: increased rates of complete cytoreduction (33% to 62%) and median OS (26 to 45 months) [12]. However, a Swedish population-based cohort study (a ‘real-world’ study) showed that whilst ultra-radical debulking improved the complete resection rate from 37% to 67%, there was no improvement in OS even when complete tumor excision was accomplished [13]. While most short-term morbidities are predictable and treatable, one study has reported that it took six to nine months for quality of life to return to preoperative levels after ultra-radical surgery [14]. This possibility should be considered when reporting the results of ultra-radical surgery. 

## 5. Non-Surgical Primary Treatment

A single small study has addressed treatment with chemotherapy alone in patients with advanced ovarian cancer [15]. All patients had tumor masses that were >2 cm. Only diagnostic or palliative surgical procedures were carried out. Twenty-nine patients received chemotherapy which was platinum-based in twenty-five patients. Overall median survival was 16 months, but was 29 months in 11 patients (35%) that achieved a complete response. These results are similar to those associated with a primary surgical approach. The authors concluded that ‘avoiding multi-organ resection does not adversely impact on survival and concerns proceeding with a prospective randomized trial of primary debulking surgery are unfounded’. 

Surgical complication rates will inevitably accompany increasingly ultra-radical surgery. Without evidence-based data, the true role of primary ultra-radical debulking surgery will remain undefined. Some patients may be better managed by radical debulking, others by NACT/IDS. Some might benefit from surgery at relapse, but in some, this should not be considered. In some patients, chemotherapy alone can lead to a long progression-free survival. It is likely that some patient subgroups might not benefit from ultra-radical surgery. Until studies are designed and completed to investigate which treatments are best for different sub-groups of patients, precise assignments of treatment will not be possible. Generalized dogmatic approaches to debulking surgery need to be addressed in order to determine if the risks and costs that are associated with outcomes (improved survivals and cures) are sufficiently improved so as to justify their utilization.

## 6. 2022 and Beyond—Interpretations and Extrapolations by the Academic Editor

### 6.1. Genomics: Markers and Targets

Both the present and future are locked onto genomics. The present status of genomics at any time in the United States is available through monthly updates from the National Comprehensive Cancer Network (NCCN) and can be summarized as “Patients with ovarian cancer, fallopian tube cancer, or primary peritoneal cancer should have genetic risk evaluation and germline and somatic testing [16]”. Germline testing refers to using blood or saliva for testing the genes that are expected to be identical throughout the body, while somatic testing focuses on the genes in a tumor and may impact treatment. Paired germline and somatic testing yield the best information as to genetic differences specifically related to a cancer. The aim at my institution is paired testing on all ovarian cancers and in reality, is nearly 100% at present. As of the beginning of 2022, germline testing related to ovarian cancer involved 36 genes: APC, ATM, AXIN2, BARD1, BRCA1, BRCA2, BRIP1, BMPR1A, CDH1, CDK4, CDKN2A, CHEK2, DICER1, EPCAM, GREM1, HOXB13, MLH1, MSH2, MSH3, MSH6, MUTYH, NBN, NF1, NTHL1, PALB2, PMS2, POLD1, POLE, PTEN, RAD51C, RAD51D, RECQL, SMAD4, SMARCA4, STK11, TP53, and somatic tumor testing involved 11 genes: ATM, BARD1, BRCA1, BRCA2, BRIP1, CHEK2, MRE11A, NBN, PALB2, RAD51C, and RAD51D with microsatellite instability testing for 6–13 markers [17,18]. Often determinations can be performed on formalin-fixed paraffin-embedded samples. Testing may be carried out in-house, but outsourcing is also possible with turnaround times of 4–6 weeks. Testing options vary from testing platform to testing platform with regard to the number of genes that can be assessed: FoundationOne^®^ CDx—interrogates 324 DNA genes from a tissue sample [19], CARIS^®^ MI Profile—interrogates 592 genes from a tissue sample* [20], Tempus xT—interrogates 648 DNA genes from a tissue sample, blood or saliva* [21], FoundationOne^®^ Liquid CDx—interrogates 324 DNA genes from a blood draw [22], Guardant360^®^—interrogates 74 DNA genes from a blood draw [23], Tempus xF—interrogates 105 DNA genes from a blood draw* [24]. (*Certain platforms do not have US Food and Drug Administration approval at the time of this writing). Genomics can identify the potential for familial risk of ovarian cancer or indicate the presence of clinically actionable drug targets. For example, the PARP inhibitor olaparib may be actionable with germline or somatic mutations for BRCA1 or BRCA2 in advanced and metastatic ovarian cancers [25,26] and pembrolizumab might be actionable in PD-L1-positive cases of ovarian cancer progression or those with microsatellite instability in advanced and metastatic ovarian cancers [27,28,29]. Currently, the genes that must be considered by the practicing physician present a complex challenge that is met by tumor boards addressing how to treat specific patients with a multidisciplinary precision medicine team approach [30], especially since individuals often have more than one actionable drug target and genomic differences occur between histological subtypes of ovarian cancer [31]. In addition, genomic variants may vary by geographic location [32] or ethnicity [33] so that newcomers to a population may express different variants from the native population. In particular, the identification of 1088 BRCA variants distinct to Chinese individuals indicates that current Caucasian population-based BRCA data are inadequate for extension to non-Caucasian groups [33]. An expectation for the future is that additional genes and variants will be involved in genomic testing and that more actionable targets will be identified that result in durable drug responses contributing to increased survival. Additional focus on genomic markers that personalize surgical radicality in order to optimize outcomes can be expected as part of new discoveries [34]. In light of this ongoing expected expansion of genes involved in ovarian cancer risk and treatment, tools that involve artificial intelligence [35] will be very helpful for sorting through genomic information so that treatment choices have the greatest odds of being successful.

It can be asked if the considerations involved in genomic analyses are destined to become too great and if guidelines could be used to focus on groups that would be the best served by genomic analyses. Recent work comparing universal testing with targeted testing based on NCCN guidelines [36] found that universal genetic testing detected more clinically actionable variants than the guideline-based approach [37], thereby arguing against a guideline-directed approach. An explanation for these findings is that the guideline-directed approach relies on self-reporting which can underestimate the applicability of individuals since 50% of BRCA carriers did not report family history risk or Jewish ancestry [38]. From the standpoint of universal population genomic testing, the two biggest barriers are cost and actions [39]. With regard to universal testing for BRCA1/2 variants in the at-risk population, under-utilization is generally reported for the US [40,41] and the UK [42]. It is noteworthy that universal BRCA testing has been reported to be cost-effective [43,44,45]. 

### 6.2. Liquid Biopsy

In recent years there have been considerable efforts to develop a blood test that could detect multiple cancers. Ovarian cancer detection would benefit if early-stage disease or early recurrence could be identified by such a blood test. Technology that examines circulating DNA in the blood from the turn-over of cancer cells that shed abnormal DNA into the bloodstream is being explored. A major report has identified forty-two genomic regions characterized by specific differential hypermethylation of precursor serous tubal intraepithelial carcinoma (STIC) lesions of the fallopian tube, of which 17 (40.5%) directly overlapped with high-grade serous ovarian carcinoma (HGSC)-specific differentially methylated regions [46]. Importantly, methylation at these shared loci was able to completely distinguish STIC and HGSC samples from normal and adjacent-normal specimens, suggesting a basis for the presence of both frank ovarian malignancy as well as STIC precursors. A clinical validation trial using a similar approach reported the overall sensitivity of the detection of all ovarian cancers at 83.1% and 50% for stage I ovarian cancer [47], indicating that this published approach does not provide robust detection of early-stage disease. At present, it is not known if this published approach provides information on early recurrence and if this identification would reduce mortality.

### 6.3. PARP Inhibitors

BRCA1 and BRCA2 mutations exert their effect on pathways involved in DNA break repair, making these patients’ tumors vulnerable to treatments that further damage DNA repair, such as poly(ADP-ribose) polymerase (PARP) inhibitors. Thus, BRCA1/2 mutations can identify an actionable PARP inhibitor pathway in tumors [48,49]. Initially, the PARP inhibitor olaparib was reported to be associated with a median overall survival benefit of 12.9 months compared to a placebo [50], and more recently has been reported associated with extending median progression-free survival beyond 4.5 years following 2 years of maintenance therapy in advanced ovarian cancers bearing a BRCA mutation [51]. When olaparib was combined with bevacizumab in the treatment of newly diagnosed stage III and IV ovarian cancers, a substantial progression-free survival benefit was reported for HRD-positive patients with a reduction of risk of progression or death of 61% in the higher-risk group (stage III with residual disease or had neoadjuvant chemotherapy or stage IV disease) and of 85% in the lower-risk group (stage III disease with a complete resection) compared with bevacizumab alone over 15 months of treatment and ~40 months of follow-up [52]. This work indicates that combination therapy with PARP inhibitors appears to have a future. A major advance to PARP therapy will be the development of methods for improving the quality of life for women on PARP inhibitors so that they can remain on these therapies longer. In addition, genomic regions of homologous recombination deficiency are reported to be predictive of sensitivity to the PARP inhibitor rucaparib exclusively in platinum-sensitive disease so that BRCA1 methylation, the RAS, AKT, and cell cycle pathways may be additional predictors of PARP inhibitor sensitivity [53,54]. Elaborating PARP inhibitors with improved activity and less toxicity is expected to occur as well as the discovery and utilization of agents such as novobiocin that may be useful alone or in combination with PARP inhibitors for treating homologous recombination-deficient tumors, including those with acquired PARP inhibitor resistance [55]. 

### 6.4. Immunotherapy

Two decades ago, it was reported that the presence of either CD3+ or CD8+ tumor-infiltrating lymphocytes in epithelial ovarian cancer was associated with improved overall survival and increased tumor expression of interferon-gamma and other lymphocyte-attracting cytokines indicating that the immune system was active against ovarian cancer [56]. However, the utilization of immune checkpoint inhibitors that have proved successful against a variety of diverse solid tumors has not been successful against ovarian cancers [57,58,59,60,61]. It is possible that this lack of success can be explained by an immunosuppressive environment in ovarian cancer. Elevated disease burden or presence of liver metastases associated with ovarian cancer are also coordinated with inferior outcomes to immune checkpoint inhibitor therapy across multiple tumor types and may also be contributing to failures in immunotherapy in ovarian cancers. The need to delineate multiple immune cell markers specific to ovarian cancers may lead to improved immunotherapies for ovarian cancers through better selection [62]. It could also be helpful to find ways to exploit subsets of immune cells that would be effective against ovarian cancers [63], including macrophages [64,65,66]. Lastly, solutions may be in combination immunotherapies as indicated by the use of pembrolizumab with bevacizumab and oral cyclophosphamide that reported an objective response rate of 47.5%, and a durable treatment response of 25% in 40 recurrent patients showing a median progression-free survival of 10 months [67].

### 6.5. Chimeric Antigen Receptor (CAR) T Cell Therapy

CAR T cell therapies have been remarkably effective in a subset of hematological malignancies that had few therapeutic options. Therapy with CAR T cells has been unsuccessful against ovarian cancers and solid tumors generally due to a lack of highly tumor-specific antigens to the target, leading to the destruction of non-tumor tissue and resulting in life-threatening “on-target/off-tumor” toxicities. T cell entry into solid tumors has also been a problem. Recently, alkaline phosphatase placental-like 2 (ALPPL2) has been identified as a tumor-specific antigen expressed in ovarian cancer and performed successfully using synthetic Notch CAR-T cells, acting as a sole target in murine models [68]. This approach holds promise but awaits clinical exploration.

### 6.6. Surgery for Interval Debulking and Recurrent Ovarian Cancer

Two trials have resolved many of the doubts about secondary cytoreduction combined with chemotherapy as compared to chemotherapy alone by achieving improved survivals of up to two additional years [6,69]. These successes lead to the possibility that additional surgeries with chemotherapy may yield survival benefits. The key to improved surgical successes is the completeness of ovarian cancer that is removed [70]. To achieve this type of success, surgeons will need new ways to visualize small deposits of tumor that otherwise might evade detection and removal. A phase III trial has demonstrated that pafolacianine sodium injection (OTL38) with near-infrared fluorescence imaging intraoperatively identified additional cancer that was not planned for resection in a statistically significant number of ovarian cancer patients [71]. Candidates for primary cytoreduction, interval debulking, or surgery for recurrent disease can benefit from technologies that identify malignant lesions that are not identifiable in normal white light. Neoadjuvant chemotherapy is used to shrink ovarian tumors before surgery. Diffusion-weighted magnetic resonance imaging can determine site-specific responses to neoadjuvant chemotherapy [72]. By doing so, the surgeon can be given assessments of tumor shrinkage, residual tumor volume, and tumor necrosis. Linking this information to robotic surgical platforms in the future may lead to more complete removal of primary, metastatic, and recurrent disease. It should not be overlooked that gynecologic oncology surgeons tend to have urban practices so that a challenge for the future is delivering care to rural women [73]. Solutions involving telemedicine and referrals with transportation will be necessary in order to offer quality care to women residing in rural areas. 

### 6.7. HIPEC

In conjunction with cytoreductive surgery, hyperthermic intraperitoneal chemotherapy (HIPEC) holds promise for treating malignancies that affect the peritoneal surface, especially microscopic residual tumors [74]. HIPEC can deliver higher concentrations of chemotherapy directly within the peritoneal cavity than can be achieved with intravenous delivery and has less side effects. Tissue penetration is increased by hyperthermia, and evidence is building for its use for both primary and recurrent ovarian cancers. HIPEC will be most effective for achieving a survival benefit when all gross and microscopic disease can be resected and suitable chemotherapy, such as cisplatin, can be used. A landmark randomized controlled trial of 245 poor prognosis women with stage III ovarian cancer achieved a 4-month progression-free survival and 12-month overall survival advantage [75]. Recently, a study involving 92 women concluded that HIPEC with carboplatin was well tolerated but did not result in superior clinical outcomes in terms of an absence of disease progression at 24 months [76]. However, with patients who had at least stage III disease with <2.5 mm of residual disease at the end of surgery and who received HIPEC, it was concluded that HIPEC was feasible in 35% and should be offered [77]. Selection for HIPEC is important with resectable disease, a peritoneal cancer index <21, and the absence of distant metastasis emerging as important factors among experts [77]. Certainly, improved cytoreduction will be key to delivering the promise of HIPEC in the future. Areas for future exploration include identifying the best chemotherapy combinations, as well as optimizing the HIPEC procedure with regard to administration, and finally integration of HIPEC with subsequent therapy. In addition, the feasibility of adding immunotherapy in a pre-adjuvant step might prove effective for treating sub-surface remainders of disease.

### 6.8. Maintenance Therapy during Recurrence

There are considerable challenges to be faced in treating women with recurrent disease. Bevacizumab has been approved in combination chemotherapy either in first-line therapy or for patients with recurrent disease not previously treated with the same drug. Recently the value of continuing bevacizumab beyond progression after first-line treatment with the same drug has been examined. Compared with standard chemotherapy alone, continuing bevacizumab beyond progression combined with chemotherapy in patients with platinum-sensitive recurrent ovarian cancer did improve progression-free survival modestly [78]. Following 2 years of maintenance therapy in advanced ovarian cancers bearing a BRCA mutation, the PARP inhibitor olaparib achieved a median of progression-free survival that was greater than 4.5 years [51]. The key to this result is enabling women to remain on PARP therapy which in many cases is challenging. Especially challenging is the frail patient for whom agent-reduction has been reported to result in poorer survival [79]. Clinical effectiveness aside, it was recently reported that none of the currently used maintenance strategies was cost effective in the US using a willingness to pay threshold of USD $100,000 [80].

### 6.9. Prevention

Opportunistic salpingectomy performed as an alternative to tubal ligation at the time of hysterectomy can prevent cancers originating in the fallopian tubes, may result in savings of USD 445 million each year, and may save 1854 deaths from ovarian cancer annually in the United States [81]. Opportunistic salpingectomy at the time of cesarean delivery has replaced tubal ligation as the most common type of sterilization; however, it is associated with higher surgical morbidity than bilateral tubal ligation [82]. Risk reduction for women who carry BRCA1/2 variants can achieve a 95% reduced risk of primary peritoneal cancer through risk-reducing prophylactic bilateral salpingo-oophorectomy [83]. 

Pregnancies are associated with a decreased risk of ovarian cancer. Compared to nulliparous women, those with 1 live birth have an approximate 24% decrease in risk of ovarian cancer, while women with two or more live births have an approximate 42% risk reduction [84]. This protection extends even to incomplete pregnancies where a 20% decreased risk results in women with ≥2 two incomplete pregnancies and improves to a 60% decreased risk for women who in addition have ≥3 complete pregnancies [85]. When oral contraceptive use is considered ever users had almost a 30% reduction in risk compared to women who never used contraceptives and risk decreased as the time of use was extended [86]. Moreover, the protective effect remained significant for up to 35 years after the last oral contraceptive use. This protective effect was also recently reported with BRCA1/2 carriers to be a 76% reduction in ovarian cancer after >10 years of oral contraceptive use and persisted for >15 years [87].

## 7. Special Issue on Ovarian Cancer 3.0—Focusing on Factors Related to the Diagnosis of Ovarian Cancers

(a) *Comparison of HE4, CA125, ROMA*, *and CPH-I for Preoperative Assessment of Adnexal Tumors* [88]. This study assessed the performance of CA125, HE4, ROMA, and CPH-I on the specific detection of epithelial ovarian cancer and metastatic carcinoma of the ovary so that these cases can be operated on by surgeons specialized in gynecologic oncology. In addition, this work evaluated the role of CA125, HE4, ROMA, and CPH-I to diagnose epithelial ovarian cancer and metastatic carcinoma of the ovary in three challenging situations: premenopausal women, stage I epithelial ovarian cancer, and adnexal masses with an inconclusive diagnosis of malignancy by ultrasound features, using IOTA simple rules.

(b) *Significance of Pelvic Fluid Observed during Ovarian Cancer Screening with Transvaginal Sonogram* [89]. This work examined the frequency and duration of free fluid during transvaginal ultrasonography in 48,925 women undergoing 326,998 ultrasound exams and correlated the findings with the diagnosis of ovarian cancers. These efforts determined that the additional information of free fluid in ultrasound findings predicts ovarian malignancy better than ultrasound alone.

(c) *Ultrasonographic Visualization of the Ovaries to Detect Ovarian Cancer According to Age, Menopausal Status and Body Type* [90]. This study examined the influence of age, menopausal status, weight, and BMI on transvaginal ultrasonographic visualization of the ovaries in 29,877 women who had both ovaries visualized on their initial exam and determined that one or both ovaries could be visualized in two of every three women over 80 years of age. Consequently, transvaginal ultrasonographic imaging should be considered viable for elderly women, and age should not be used to deny access to TVS.

(d) *Salvaging Detection of Early-Stage Ovarian Malignancies When CA125 Is Not Informative* [91]. This investigation evaluated the ability of the OVA1 multivariate assay to salvage the detection of ovarian cancers in 2305 women when CA125 had been non-informative due to low or “normal” serum values. OVA1 successfully identified 59% of pelvic malignancies and 63% of early-stage ovarian cancers that were missed by serum CA125 alone. OVA1 can identify ovarian malignancy despite normal serum CA125 findings so that expeditious referral to a gynecologic oncologist can lead to appropriate treatment and improved overall survival.

(e) *Detection of Tumor-Specific PTPmu in Gynecological Cancer and Patient-Derived Xenografts* [92]. This report used the peptide agent SBK4 conjugated to the fluorophore Texas Red to label tumor tissue microarrays containing patient and/or patient-derived xenograft samples from several high-grade ovarian cancers and quantified the level of staining. These efforts were able to directly compare the patient and the matched patient-derived xenograft tissue on the same slide.

(f) *Diagnostic Added-Value of Serum CA-125 on the IOTA Simple Rules and Derivation of Practical Combined Prediction Models (IOTA SR X CA-125)* [93]. This investigation evaluated the diagnostic value of adding serum CA-125 to the IOTA Simple Rules ultrasonographic evaluation to differentiate between malignant and benign ovarian tumors before surgery in 479 women. This work found that serum CA-125 significantly increased the predictive value in combination with the IOTA Simple Rules in differentiating malignant adnexal masses from benign.

(g) *Low-Grade Serous Carcinoma of the Ovary: The Current Status* [94]. This review focuses on the unique characteristics of low-grade serous carcinoma of the ovary including tumorigenesis/histogenesis, genomics, pathology grading, immunohistochemistry, tumor markers, and appearance during ultrasound, computed tomography, magnetic resonance imaging, and positron emission tomography. It also considers mutational status, hormone receptor status, proliferation index, management, and future directions.

(h) *Ovarian Carcinosarcoma with Retroperitoneal Para-Aortic Lymph Node Dissemination Followed by an Unusual Postoperative Complication: A Case Report with a Brief Literature Review* [95]. This case report and review focuses on ovarian carcinosarcoma, also known as malignant mixed Müllerian tumor, which is one of the rarest histological subtypes of ovarian cancer and has a dismal prognosis. The authors concluded that retroperitoneal, pelvic, and para-aortic lymph nodes should be closely inspected because retroperitoneal para-aortic lymph node metastasis could be the only extrapelvic dissemination of ovarian carcinosarcoma. They state that there are limited data on etiology, diagnosis, prognostic factors, and treatment of ovarian carcinosarcoma, while some studies concluded that prognostic factors and treatment of carcinosarcomas are associated with epithelial components because carcinosarcomas are carcinomas with epithelial–mesenchymal transition and heterologous differentiation.
